# Treatment outcome of smear-positive pulmonary tuberculosis patients in Tigray Region, Northern Ethiopia

**DOI:** 10.1186/1471-2458-12-537

**Published:** 2012-07-23

**Authors:** Gebretsadik Berhe, Fikre Enquselassie, Abraham Aseffa

**Affiliations:** 1 College of Veterinary Medicine, Mekelle University, Mekelle, Ethiopia; 2 School of Public Health, Addis Ababa University, Addis Ababa, Ethiopia; 3 Armauer Hansen Research Institute, Addis Ababa, Ethiopia

**Keywords:** Smear-positive, Treatment outcome, Pulmonary tuberculosis, Tigray, Ethiopia

## Abstract

**Background:**

Monitoring the outcome of tuberculosis treatment and understanding the specific reasons for unsuccessful treatment outcome are important in evaluating the effectiveness of tuberculosis control program. This study investigated tuberculosis treatment outcomes and predictors for unsuccessful treatment outcome in the Tigray region of Ethiopia.

**Methods:**

Medical records of smear-positive pulmonary tuberculosis (PTB) patients registered from September 2009 to June 2011 in 15 districts of Tigray region, Northern Ethiopia, were reviewed. Additional data were collected using a structured questionnaire administered through house-to-house visits by trained nurses. Tuberculosis treatment outcomes were assessed according to WHO guidelines. The association of unsuccessful treatment outcome with socio-demographic and clinical factors was analyzed using logistic regression model.

**Results:**

Out of the 407 PTB patients (221 males and 186 females) aged 15 years and above, 89.2% had successful and 10.8% had unsuccessful treatment outcome. In the final multivariate logistic model, the odds of unsuccessful treatment outcome was higher among patients older than 40 years of age (adj. OR = 2.50, 95% CI: 1.12-5.59), family size greater than 5 persons (adj. OR = 3.26, 95% CI: 1.43-7.44), unemployed (adj. OR = 3.10, 95% CI: 1.33-7.24) and among retreatment cases (adj. OR = 2.00, 95% CI: 1.37-2.92) as compared to their respective comparison groups.

**Conclusions:**

Treatment outcome among smear-positive PTB patients was satisfactory in the Tigray region of Ethiopia. Nonetheless, those patients at high risk of an unfavorable treatment outcome should be identified early and given additional follow-up and social support.

## Background

Despite the availability of highly effective treatment for decades, tuberculosis (TB) remains a major global health problem. In 2010, there were an estimated 8.5–9.2 million new cases and 1.2–1.5 million deaths worldwide [1]. The foundation of the current global TB strategy began in the 1990s, when the increasing trends of TB led to the creation of directly observed treatment- short course (DOTS) strategy. The multidimensional DOTS framework has been implemented in 184 countries and over 132 million patients have been treated with DOTS resulting in more than 125 million being cured [2-5]. The specific targets of DOTS detailed in the updated Global Plan (2011–2015) are to achieve a case detection rate (CDR) of 84% (for all cases and smear-positive cases specifically) and a treatment success rate (TSR) of 87% by 2015 [6].

According to the WHO Global TB report 2011, Ethiopia ranks 8^th^ in the list of 22 high burden countries (HBCs), and 3^rd^ in Africa, with an estimated prevalence of all forms of TB in 394 per 100,000 population [1]. TB is the leading cause of morbidity, the third cause of hospital admission, and the second cause of death in Ethiopia [7]. Ethiopia started implementing DOTS within a standardized TB prevention and control program in 1992 [7]. Currently, Ethiopia reports treatment success and case detection rates of 83% and 72% of all forms of TB, respectively. DOTS coverage is estimated at 100% geographical and 95% health facility level [8].

The Tigray region in Northern Ethiopia initiated DOTS program in 1995 [9]. The Region has an estimated population of 4.8 million, with a TB case notification rate of 240 cases/100,000 population and a DOTS geographical coverage rate of 100%. There were 168 functional TB diagnostic facilities in the region in 2010 [10]. The DOTS program has been introduced in all hospitals, health centers and in most health posts in the Region. The direct observation of TB treatment has been decentralized from hospitals and health centers to health posts [9,11]. According to the Regional Health Bureau report, among smear-positive pulmonary tuberculosis (PTB) cases evaluated in 2009, 4.6% died, 1.5% defaulted and 0.8% failed contributing to a total of 2.7% unfavorable outcome [10].

Monitoring the outcome of treatment is essential in order to evaluate the effectiveness of the DOTS program [12]. Furthermore, understanding the specific reasons for unsuccessful outcomes is important in order to improve treatment systems [13]. In this regard, studies in some parts of Ethiopia- Southern region [14] and Gondar area [15] reported 74.8% and 29.5% treatment success rates in TB patients, respectively. These and various other studies in Southern region [14,16], Arsi zone [17], Gondar area [15], as well as Addis Ababa area of Ethiopia [18] have documented independent risk factors for poor treatment outcome. These factors include attending the regional capital health centre, being on retreatment, having a positive smear at the second month follow-up, age being more than 55 years, being male, medication side effects, low body weight at initiation of anti-TB treatment (<35 kg), year of enrollment, distance from home to treatment centre and the added burden of using public transport to get to a treatment centre.

Despite the high DOTS region-wide coverage and the progress made in TB control in the Tigray region of Ethiopia, the treatment outcome of TB patients has not been assessed so far. There is little information on what factors are responsible for unsuccessful treatment outcome in the Region. In this study, we assessed the treatment outcomes of smear-positive PTB patients on DOTS and identified factors associated with un-successful outcome in the Tigray region of Ethiopia.

## Methods

### Study area

**Figure 1:**
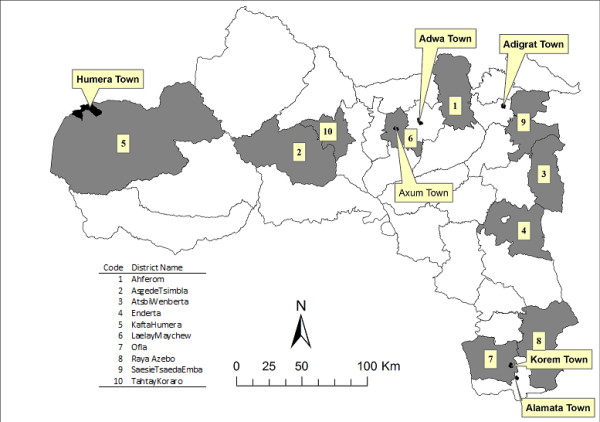
Rural and urban study districts in Tigray region, Northern Ethiopia.

The study was carried out in Tigray region, Northern Ethiopia. Ten rural and five urban districts in the five zones (Southern, Eastern, Central, North Western and Western zones) of the Region were included in the study. Data were collected from all health centers located in Atsbi-Wenberta, Saesie-Tsaedaemba, Enderta, Tahtay-Koraro, Laelay-Maichew, Raya-Azebo, Adwa, Offla, Asgede-Tsimbla, Setit-Humera, Kafta-Humera, Korem, Adigrat, Ahferom, and Axum districts (Figure 1).

### Study design and data collection

The determinants of treatment outcome were assessed through retrospective and cross-sectional study designs. A retrospective analysis was conducted on the profile and treatment outcome of all smear-positive PTB patients registered from September 2009 to June 2011 at all DOTS facilities in the 15 selected districts. The reviewed documents contained basic information such as patient's age, sex, address, TB type, treatment category, HIV status and treatment outcome. Additional information was collected using a structured questionnaire through house-to-house visit of PTB patients who were identified in a review of medical records. In addition to the information in the TB Registry, we collected data on income, educational status, family size, religion, ethnicity, and distance from treatment centre from all enrolled PTB patient.

Data were collected by trained nurses. The study focused on smear-positive PTB patients because smear-positivity results from harboring a highly contagious form of *M. tuberculosis* and can be monitored for speed of bacteriologic conversion on chemotherapy [19,20].

### Sample size and sampling

In this study, sample size was calculated considering the “proportion of smear-positive PTB patients with unfavorable treatment outcome” as a predictor variable. Sample size was determined using single population proportion formula. The following parameters were taken into account during calculation of sample size: prevalence of unfavorable outcome of 2.7% [10], 95% confidence interval and a maximum discrepancy of + 3% between the sample and the underlying population; then the result was multiplied by 3 to consider the cluster effect and increase power. Thus, a minimum number of 336 study subjects were required.

Multistage cluster sampling technique was used to randomly select the different districts. Hence, ten rural and five urban districts were selected by simple random sampling method and smear-positive PTB patients from the selected districts were recruited consecutively during the limits of study period at these sites.

### Exposure assessment and outcome definition

#### Exposure assessment

For each identified patient who underwent TB therapy under DOTS, the following information was collected from the medical records and the administered additional questionnaire: age, sex, family size, religion, ethnicity, place of residence, educational status, occupation, treatment category, HIV status, distance from treatment center and treatment outcome. For defaulters, time and reasons for defaulting were also recorded.

#### Outcome definition

TB treatment outcome categories were defined according to WHO and the International Union Against Tuberculosis and Lung Disease guideline [21]. WHO defines treatment success as the sum of patients who are cured and those who have completed treatment. In line with WHO criteria, treatment outcomes were categorized into:

a. Successful outcome- if PTB patients were cured (i.e., negative smear microscopy at the end of treatment and on at least one previous follow-up test) or completed treatment with resolution of symptoms.

b. Unsuccessful outcome – if treatment of PTB patients resulted in treatment failure (i.e., remaining smear-positive after 5 months of treatment), default (i.e., patients who interrupted their treatment for two consecutive months or more after registration), or death.

However, patients who transferred out to other districts were excluded from the treatment outcome evaluation as information on their treatment outcome was unavailable.

### Statistical analysis

We used STATA Version 10.0 for windows program (STATA Corp, College Station, Texas, USA) for data analysis. Relationships between treatment outcomes and potential predictor variables were assessed using bivariate and multivariate logistic regression model. The age, sex, family size, place of residence, educational status, employment status, treatment category, HIV status and distance from treatment centers of PTB patients were subjected to multivariate analysis and the final model was determined with enter method.

### Ethical consideration

This study was approved by the respective institutional review boards at College of Health Science, AAU and the Armauer Hansen Research Institute. Written informed consent was obtained from all study participants.

## Results

### Socio-demographic and clinical factors

A review of the treatment records of 407 smear-positive PTB patients was retrieved from all health centers found in the study districts. Atsbi-Wenberta district had the highest number of participants (12.0%) while Axum and Enderta districts had the lowest number (both 1.2%) (Table 1).

**Table 1:** Distribution of study participants by districts in Tigray region of Ethiopia

**S. No.**	**Districts**	**Residence type**	**No. of observations**	**Percent**
1	Adigrat town	Urban	23	5.65
2	Adwa town	Urban	25	6.14
3	Ahferom	Rural	41	10.07
4	Asgede Tsimbla	Rural	40	9.83
5	Astbi Wenberta	Rural	49	12.04
6	Axum town	Urban	5	1.23
7	Enderta	Rural	5	1.23
8	Humera town	Urban	40	9.83
9	Kafta Humera	Rural	23	5.65
10	Korem town	Urban	25	6.14
11	Laelay Maichew	Rural	12	2.95
12	Offla	Rural	40	9.83
13	Raya Azebo	Rural	11	2.70
14	Saesie Tsaedaemba	Rural	38	9.34
15	Tahtay Koraro	Rural	30	7.37
		**Total**	**407**	**100.00**

Among the 407 patients enrolled in the study, 221 (54.3%) were males and 186 (45.7%) females (Table 2). The mean age and standard deviation (SD) of study subjects was 36.9 ± 15.3 (range: 15–82) years. Family size varied from 1–10 persons (mean ± SD = 4.25 ± 2.0). Income distribution among the study population showed that 62.4% of the enrolled participants had less than 300 Birr monthly income while 28.8% had between 300–999 Birr income per month. Concerning educational status, 55.0% of the study participants were illiterate, 25.6% had completed primary education, and 19.4% had completed high-school and above. Occupationally, the patients comprised 127 (31.2%) unemployed, 242 (59.5%) unskilled workers, and 38 (9.3%) skilled workers. With regard to HIV status, 8.6% of the patients were sero-positive (Table 3).

**Table 2:** Treatment outcomes of smear-positive PTB patients by age and sex in Tigray region, Ethiopia

**Characteristics**	**Total (n = 407)**	**Cured**	**Treatment completed**	**Death**	**Treatment failure**	**Defaulted**	**Transfer out**
**Age (years)**							
15-24	99	84	4	4	4	2	1
25-34	99	86	2	2	3	3	3
35-44	88	74	4	3	3	4	0
45-54	53	41	6	2	2	2	0
55-65	39	30	1	3	3	1	1
65+	29	24	1	2	0	1	1
**Sex**							
Female	186	160	8	4	6	6	2
Male	221	179	10	12	9	7	4

**Table 3:** Socio-demographic and clinical characteristics of smear-positive PTB patients in Tigray region, Ethiopia

**Characteristics**	**Frequency**	**Percent**
**Age (years)**		
15-40	253	62.2
>40	154	37.8
**Sex**		
Female	186	45.7
Male	221	54.3
**Family size**		
1-5	262	64.4
>5	145	35.6
**Religion**		
Orthodox Christians	384	94.35
Others	23	5.65
**Ethnic group**		
Tigrawai	386	94.9
Amhara	16	3.9
Afar	5	1.2
**Residence**		
Urban	196	48.2
Rural	211	51.8
**Educational status**		
Illiterate	224	55
Elementary	104	25.6
High school	62	15.2
College	17	4.2
**Occupation**		
Unemployed	127	31.2
Unskilled worker	242	59.5
Skilled worker	38	9.3
**Income (Birr)**		
< 300	254	62.4
300-999	117	28.8
> 1000	36	8.8
**Treatment category+**		
New cases	379	94.5
Re-treatment cases	22	5.5
**HIV status**		
Positive	35	8.6
Negative	271	66.6
Unknown	101	24.8
**Distance to treatment center+**		
<= 10 km	219	54.6
> 10 km	182	45.4

+The totals add up to 401 (Transfer out cases were not included).

### Treatment outcomes and factors affecting the outcomes

Among the PTB patients enrolled in this study, 343 (85.5%) were cured, 18 (4.4%) had completed their treatment and 6 (1.47%) were transferred out. From the 401 patients evaluated for treatment outcome, 357 (89%) had successful and 44 (10.8%) unsuccessful outcomes. Of the patients with unsuccessful treatment outcome, 15 (3.7%) had treatment failure, 13 (3.2%) had defaulted and 16 (3.9%) had died (Table 2).

Bivariate and multivariate logistic regression analysis was carried out for selected socio-demographic and clinical risk factors including age, sex, family size, place of residence, educational status, employment status, treatment category of patients, HIV status and distance from treatment centers. In the final multivariate logistic model, the proportion recorded as having an unsuccessful treatment outcome varied by age group, family size, employment status and treatment category (Table 4). The risk of unsuccessful treatment outcome was 2.5 (95% CI: 1.12-5.59) times higher among PTB patients older than 40 years of age compared to those aged 15–40 years. Compared to PTB patients having 1–5 family size, those PTB patients having family size greater than 5 persons had 3.3 (95% CI: 1.43-7.44) times greater risk of unsuccessful treatment outcome. Unemployed PTB patients were more likely to experience (adjusted OR = 3.10, 95% CI: 1.33-7.24) unsuccessful outcome when compared to their counterparts. Unsuccessful treatment outcome was more frequent (adjusted OR = 2.00, 95% CI: 1.37-2.92) among retreatment cases than among those newly treated. Sex, residence type, educational status, HIV status and distance from treatment center of PTB patients did not show any statistically significant association with unsuccessful treatment outcome in the multivariate analysis (Table 4).

**Table 4:** Logistic regression analyses of factors associated with treatment outcome in smear-positive PTB patients in Tigray region, Ethiopia

**Characteristics**	**Treatment outcome**			
	N*	**Unsuccessful n (%)**	**COR (95% CI)**	**AOR (95% CI)**
**Age (years)**				
15-40	249	18 (7.2)	1.00	1.00
>40	152	26 (17.11)	1.25(1.39-5.02)	**2.50 (1.12-5.59)**
**Sex**				
Female	184	18 (9.78)	1.00	1.00
Male	217	26 (11.98)	1.25 (0.66-2.37)	0.97 (0.44-2.15)
**Family size**				
1-5	259	21 (8.11)	1.00	1.00
>5	142	23 (16.19)	2.19 (1.16-4.11)	**3.26 (1.43-7.44)**
**Residence**				
Urban	193	20 (10.36)	1.00	1.00
Rural	208	24 (11.54)	1.13 (0.60-2.11)	0.70 (0.29-1.67)
**Educational status**				
Formal education	170	12 (7.06)	1.00	1.00
Illiterate	231	32 (13.85)	2.12 (1.06-4.24)	2.36 (0.95-5.85)
**Employment**				
Employed	276	26 (9.42)	1.00	1.00
Unemployed	125	18 (14.40)	1.62 (0.85-3.07)	**3.10 (1.33-7.24)**
**Category of treatment**				
New smear positive	379	36 (9.62)	1.00	1.00
Re-treatment cases	22	8 (36.36)	1.75 (1.28-2.39)	**2.00 (1.37-2.92)**
**HIV status**				
Negative	268	29 (10.82)	1.00	1.00
Positive	35	7 (20,00)	2.06 (0.83-5.14)	1.84 (0.63-5.39)
**Distance to treatment center**				
<= 10 km	219	25 (11.41)	1.00	1.00
> 10 km	182	19 (10.44)	0.90 (0.48-1.70)	0.96 (0.40-2.28)

N = Number of observations; COR = Crude odds ratio; AOR = Adjusted odds ratio; CI = Confidence interval.

* The total number of patients evaluated across each subgroup adds up to 401 excluding the 6 patients who were transferred out to other districts.

## Discussion

Assessment of treatment outcome and analysis of factors responsible for unsuccessful treatment outcome in DOTS programs is of paramount importance particularly in smear-positive PTB patients as they harbor a highly contagious form of *M. tuberculosis* that can be monitored for speed of bacteriologic conversion on chemotherapy [19,20].

In this study, treatment success in smear-positive PTB patients was 89.0%, slightly higher than the WHO international target of 87% (updated target 2011–2015) but remarkably higher than previous studies conducted in some parts of Ethiopia including 74.8% in Southern region [14] and 29.5% in Gondar area of Ethiopia [15]. A recent community-randomized trial intervention in Southern Ethiopia has also reported a similarly high proportion of successful outcome (89.3%) using health extension workers (HEWs) to follow-up the patients [22]. Our finding of higher successful outcome in Tigray region as compared to other areas in Ethiopia could be the result of the decentralization of DOTS to health posts in Tigray region that has substantially reduced treatment default from 32% in 1996 to 15% in 2003 [9]. According to the report, this was attributed to two main factors: health posts nearer to patients' residence and the use of volunteer community health workers (CHWs) in tracing patients who default from treatment [9]. This is also consistent with a finding in Tanzania where community based DOTS had higher successful outcome rate (81%) as compared to facility based DOTS (70%) [23]. Another likely reason for the higher successful outcome could be the 100% physical access to a treatment centre in the Region [10]. A study conducted in Addis Ababa reported that patients’ attitude and behavior towards the disease are major factors influencing treatment adherence [24]. This higher successful treatment outcome rate in Tigray region implies that DOTS performance is encouraging and the region is on the right track in achieving the WHO targets and the millennium development goals (MDG) in TB control.

The 10.8% unsuccessful outcome found in this study is comparatively lower than the 16.7% report from Southern Ethiopia [14] and the 11.3% default rate in Arsi Zone of Oromia [17]. The 3.2% default and 3.9% death rate recorded in this study is also lower when compared with the corresponding outcomes from Gondar area, Northwest Ethiopia, where 18.3% patients had defaulted and 10.1% had died [15]. Studies conducted in other parts of Ethiopia recorded higher proportion of poor outcome [14,15,17] compared to our data. This difference could be due to variation in DOTS performance in the various study areas. This could be attributed to the use of community health workers in tracing and follow-up of TB patients in Tigray region [9] and Southern Ethiopia [22] that has resulted in an improved performance of DOTS as compared to other areas that do not use this strategy. Other reasons for this variation could be the difference in duration of study period, sample size and study setting. For example, the study in Southern Ethiopia was conducted over a longer period (2002–2007) and involved more than 6547 patients. Unlike our study, the study in Gondar area, Northwest Ethiopia, was conducted in a hospital setting.

Elsewhere in Africa, different outcomes had been reported in different countries. A study conducted in Nigeria recorded 76.6% cured, 8.1% failed, 6.6% defaulted, 2% treatment interruption, 4.8% transferred out, and 1.9% died [25]. Another study in Tanzania reported treatment success rates of 81% and 70% in patients under community vs. facility-based DOTS, respectively [23]. Among the 4003 smear-positive PTB patients evaluated on DOTS in Malawi, 72% had completed treatment, 20% had died, 4% defaulted, 2% were transferred out and 1% had still positive smears at the end of treatment [26].

In a multivariate regression model, this study showed that unsuccessful treatment outcome was significantly higher among patients older than 40 years of age, family size greater than 5 persons, among those unemployed and amongst re-treatment patients, as compared to their counterparts.

Our observation of poor outcome in patients older than 40 years of age as compared to those aged 15–40 years is in agreement with the findings of previous studies in which older age increases the risk for unfavorable treatment outcome [13-15,27-30]. One study stated that an age in excess of 46 years was found to be a significant risk factor for non-successful treatment outcome [27]. Another study in Thailand showed that an age of above 60 years was significantly correlated with treatment interruption and treatment failure [29]. Higher age has been previously reported to be a risk factor for death [15,31]. It was documented that individuals at the extremes of age had the poorest outcomes [14]. Older individuals often have concomitant diseases and general physiological deterioration with age, less able to reach health facilities and are also poorer than the younger population [14,32-34].

Data from this study revealed that retreatment cases have an increased risk of unsuccessful outcome compared to new cases. This is consistent with other published reports, in which history of prior TB treatment was significantly associated with unsuccessful treatment outcome [14,18,27,29,35,36]. It is also reported that prior sub-optimal therapy is known to be a major contributor to the development of multidrug resistance (MDR) TB [37]. Thus, the high proportion of unsuccessful outcome in retreatment cases in our study could be related to a higher frequency of drug resistance. The prevalence of MDR TB in Ethiopia is estimated to be 1.6% among new cases and 12% among retreatment cases [5]. According to a previous study, risk factors for unsuccessful outcome were associated with patient behavior and attitudes, as patients registered as defaulters tend to default again [14]. Other risk factors include selection of drug-resistant strains and the development of severe and complicated forms of the disease, all of which contribute to poor outcome among previously treated patients [14].

The higher proportion of unsuccessful treatment outcome in patients with family size greater than 5 persons or those unemployed could be due to the relation of unemployment and larger family sizes to low income. Patients with low income often suffer from malnutrition which may result in more drug side effects and low stamina among patients and may possibly lead to poor adherence, death or discontinuation of anti-TB chemotherapy. A study in Estonia [38] and Brazil [39] suggested that one of the main risk factors for TB was poverty. In our study, the majority of the TB patients (62.4%) had very low family income (<300 Birr per month). In agreement with this study, another study reported that unemployment was highly associated with unfavorable treatment outcome [40].

Unlike the results of other studies, factors such as sex of patients, educational status, HIV status, and distance from treatment center did not show any statistically significant association with unsuccessful treatment outcome. According to many reports, urban residents [15,40] and women [13,15,41] had higher probabilities of successful treatment outcome.

The lack of any appreciable link between HIV status of patients and distance from treatment centre with TB treatment outcome was somewhat unexpected. Other studies had also indicated that most of the factors associated with treatment non-completion, apart from the patient’s age and level of education, are those related to physical access to health-care services [16]. These differences between this study and other study results could be explained by differences in sample size among the studies, difference in disease burden, and socio-demographic factors. Variations in environmental factors or true biological effects, or even a combination of all factors could also explain the differences in the study results. In Tigray region, access to health care services was facilitated by the community health workers and this may have contributed to improved outcome, including for the HIV co-infected patients.

Previous studies established that HIV is associated with unsuccessful treatment outcomes which include treatment interruption [29] and death [35]. As previously reported, smear-negative PTB patients had the lowest rate of successful treatment outcome [42,43]. These patients have a higher frequency of HIV co-infection; in addition, they may be less able to develop an adequate immune response to control the infection; furthermore their diagnosis is difficult, often resulting in treatment delay and poor outcome [44]. Another study conducted in Ethiopia has shown that HIV-positive patients are more likely to default than HIV-negatives [45]. This study also reported default rates of nearly 19% in extra-pulmonary TB (EPTB) and approximately 28% in smear-negative PTB (including EPTB). This and other related studies have indicated a background of HIV infection in these types of TB [45]. Thus, on the other hand, the lack of association between HIV status and unsuccessful treatment outcome observed in this study may be due to the exclusion of these forms of TB associated with HIV infection.

The strength of this study lies in its ability to collect verified data from TB patients to determine treatment outcomes. Studying 15 randomly selected districts has enabled us to generalize our findings to the Region. Otherwise, the study was partly based on retrospective design; therefore, selection bias could occur as we were unable to trace the whereabouts of some patients. Other important variables including patient-health worker communication, delay in health care seeking and provider and health system related factors were not assessed. Furthermore, treatment outcome and associated risk factors for patients with extra-pulmonary TB, those with smear-negative PTB and patients younger than 15 years of age were also not evaluated in this study.

## Conclusions

This study has demonstrated the success of DOTS program in smear-positive PTB patients in the Tigray region, Northern Ethiopia. Moreover, the following risk factors were identified as predictors of unsuccessful treatment outcome: older age, family sizes greater than 5 persons, unemployed and retreatment cases. Following this observation, we recommend that patients at high risk of unsuccessful treatment outcome should be identified early and given additional follow-up and a combination of additional medical intervention and social support.

## Competing interest

The authors declare that there is no competing interest among authors.

## Authors’ contribution

GB participated in all phases of preparation of the manuscript starting from inception of the project, collection of data, analysis and interpretation of results and writing of the manuscript and as corresponding author. FE contributed to interpretation of the data and writing of the manuscript. AA has participated in the design of the study, the interpretation of results and writing of the manuscript. All authors read and approved the final manuscript.

## Pre-publication history

The pre-publication history for this paper can be accessed here:

http://www.biomedcentral.com/1471-2458/12/537/prepub
